# High Reserve in δ-Tocopherol of *Peganum harmala* Seeds Oil and Antifungal Activity of Oil against Ten Plant Pathogenic Fungi

**DOI:** 10.3390/molecules25194569

**Published:** 2020-10-06

**Authors:** Abdelhamid Hajji, Fethi Bnejdi, Mourad Saadoun, Ibtissem Ben Salem, Imededdine Nehdi, Hassen Sbihi, Fahad A. Alharthi, Safia El Bok, Naima Boughalleb-M’Hamdi

**Affiliations:** 1 Biodiversity, Biotechnology, and Climate Change Laboratory, Tunis El Manar University, Tunis 2092, Tunisia; Fethibnejdi@yahoo.fr (F.B.); saadoun2003@yahoo.fr (M.S.); safia1elbok@yahoo.fr (S.E.B.); 2 High Institute Agronomic of Chott-Mariem, University of Sousse, Sousse 4042, Tunisia; ibtibesa82@gmail.com (I.B.S.); n.boughalleb@laposte.net (N.B.-M.); 3 Chemistry Department, College of Science, King Saud University, P.O. Box 2455, Riyadh 11451, Saudi Arabia; imed12002@gmail.com (I.N.); hmsbihi@ksu.edu.sa (H.S.); fharthi@ksu.edu.sa (F.A.A.)

**Keywords:** *Peganum harmala*, seed oil, population variability, δ-tocopherol, antifungal activity

## Abstract

This investigation included the chemical analysis of *Peganum harmala* (*P. harmala*) seed oil and its antifungal properties against 10 fungal species. Seed oils of six populations were analyzed using high performance liquid chromatography (HPLC) and gas chromatograph/mass spectrometry (GC-MS). The HPLC analysis indicated that *P*. *harmala* seed oil exhibited a very high level of tocopherol contents, with values in the range of 2385.66–2722.68 mg/100 g. The most abundant tocopherol isomer was δ-tocopherol (90.39%), followed by γ-tocopherol (8.08%) and α-tocopherol (1.14%). We discovered for the first time the presence of tocotrenols in *P. harmala* seed oils of the six populations studied. The GC-MS analyses revealed that linoleic acid was the main fatty acid (65.17%), followed by oleic acid (23.12%), palmitic acid (5.36%) and stearic acid (3.08%). We also studied the antifungal activity of seed oil of the Medenine (MD) population on ten fungal pathogens. The antifungal effects differed among pathogens and depended on oil concentrations. Seed oil of the MD population caused a significant decrease in mycelial growth of all fungi tested, with values ranging 31.50–82.11%, except for *Alternaria* sp., which showed no inhibition. The antifungal activity against the 10 selected fungi can be explained by the richness in tocols of the extracted oil and make *P. harmala* a promising crop for biological control. Furthermore, the importance of fatty acids and the wide geographic spread in Tunisia of this species make this crop a potential source of renewable energy.

## 1. Introduction

*Peganum harmala* Linn, also known as wild rue, harmel or esphand, is a perennial plant species belonging to the Nitrariaceae family (previously called Zygophyllaceae). The Nitrariaceae is a widespread family including 27 genera and 285 species subdivided into five subfamilies [1]. It consists of herbs, shrubs and trees growing in arid and semi-arid areas, in tropical and subtropical regions [2]. The plant is widely distributed in North Africa, Central Asia and the Middle East and has been introduced into America and Australia [3]. In terms of botany, *P. harmala* is a highly branched perennial shrub growing up to 1 m tall with short creeping roots [4]. The plant grows in semi-arid and arid conditions, steppe areas and sandy soils. It possesses narrow leaves, arranged alternately on fleshy, bright green and stiff stems. Flowers are solitary, small, pale yellow or white, with five petals, and the flowering period is March–April. The fruit is a capsule (about 6–10 mm wide) with three chambers. The capsules contain about 50 small black-brown triangular seeds [3].

*Peganum harmala* plays an important role in local ecosystem restoration as a drought-resistant species and is considered among the most important medicinal plants in North Africa. It is a rich source of β-carboline alkaloids (e.g., harmine, harmaline, harmalol and harman) and quinazoline alkaloids (e.g., vasicine and vasicinone) [5]. Steroids, anthraquinones, flavonoids, polysaccharides and amino acids are also found in its seeds, flowers, leaves, stems and roots [6].

Various parts of this plant have been used in folk medicine for the treatment of human ailments such as asthma, colic, lumbago, jaundice and menstrual flow [7]. Several pharmacological effects have also been reported, such as insecticidal, anti-malarial and antitumor [8]; antihistaminic, antispasmodic and vasorelaxant [9]; wound-healing, immune-modulating, leukemia-healing and antioxidant activities [10]; and hypoglycemic activities [11]. Furthermore, this plant shows analgesic, anti-inflammatory, antimicrobial and antifungal properties [12]

Recent studies investigated the pharmaceutical application, chemical composition and antimicrobial activity of essential oils extracted from seeds of *P. harmala* [13]. However, there are few reports concerning the chemical composition and microbiological activity of oils extracted from *P. harmala* seeds [14]. The present paper focuses on the tocols (i.e., tocopherols and tocotrienols) and fatty acid composition of seed oils of *P. harmala* plants, collected from different regions of Tunisia. Based on the wide geographic distribution of *P. harmala* in Tunisia with semi-arid, arid and Saharan climatic zones, we hypothesized that the geographic origin of the plant affected the tocol and fatty acid contents of the oils extracted from seeds. Various studies showed that tocols and fatty acids have many antifungal activities [15,16]. Knowledge of bio-active components would highlight the importance of traditional uses of medicinal plants and enrich the global database of phytochemicals. Plant compounds are usually influenced by geographical distribution. In this study, the principal constituents and antifungal effect of Tunisian *P. harmala* seed oil were addressed.

## 2. Results and Discussion

### 2.1. Seed Oil Content

The seed oil content and fatty acid composition were determined for six *P. harmala* populations collected from different regions in Tunisia. Data analysis indicated that seed oil content varied among localities and ranged from 5.75% to 14.75% for GF and KO, respectively, with an average of 12.51% (Table 1). These results were similar to a previous report [17], in which oil content was 10%. This finding supported the use of *P. harmala* as a new source of vegetable oil.

### 2.2. Tocol Composition

Tocols of the six *P. harmala* oils were presented in Table 1 and supplementary materials. The HPLC analyses showed that *Peganum* oils were rich on tocols. They contained four tocopherol isomers—α-, β-, γ- and δ-tocopherol (abbreviated α-T, β-T, γ-T, and δ-T, respectively)—as well as three tocotrienol isomers: α-, γ- and δ-tocotrienol (α-T3, γ-T3 and δ-T3, respectively).

All *P. harmala* oil samples contained very high amounts of tocols, ranging from 2092.78 (SE) to 3770.66 mg/100 g (MD), with an average of 2722.68 mg/100 g. The predominant isomer was clearly δ-T, representing 90% of the total tocols and an average content of 2441.78 mg/100 g (Table 2). The δ-T values ranged from 1900.51 (SE) to 3424.41 mg/100 g (MD). The γ-T was also detected in considerable levels, with contents ranging from 143.18 (SE) to 277.61 mg/100 g (MD), with an average of 205.46 mg/100 g (7.6%), whereas the α-T content varied from 19.27 (KO) to 51.05 mg/100 g (GF). The β-T was detected at lower quantities (values ranged 6.17–10.8 mg/100 g). The γ-T3 was detected at lower amount, with an average content of 17.3 mg/100 g. Previous studies [18,19,20] reported similar variability in tocol contents, possibly due to the characteristics of regions prospected. In our work, δ-T was the most abundant isomer, but in a previous study only low contents of this component were found in most tested vegetable oils (0–6% of total tocols) [21]. The search for other unconventional oils with a high δ-T content should be pursued.

The tocol contents in *P. harmala* seed oils determined in this work were very high (average content of 2722.68 mg/100 g) compared to common vegetable oils such as of eshvarak (1082.35 mg/100 g), olive (42.54 mg/100 g), sunflower (95.02 mg/100 g), soya (179.62 mg/100 g) and corn (152.94 mg/100 g) [21]. Tocols have been widely studied due to their high antioxidant, anti-aging, anticancer and anti-atherosclerosis effects [22,23].

### 2.3. Fatty Acid Contents

The major and minor fatty acid composition of the six *P. harmala* accessions are listed in Table 2. The major fatty acids identified in *P. harmala* seed oils were C16:0, C18:0, C18:1 ω7, C18:1 ω9 and C18:2; however, fatty acids, such as vaccenic (C18:1 ω7), linolenic (C18:3 ω3), arachidic (C20:0), behenic (C22:0) and tetracosanoic (C24:0) acids were detected at lower levels.

#### 2.3.1. Major Fatty Acids

Five major fatty acids were identified in the six *P. harmala* populations from Tunisia. Linoleic acid (C18:2) was the main component detected in *P. harmala* oil. Previous studies also reported that linoleic acid was the most abundant fatty acid in *P. harmala* seed oils, with contents ranging from 54.6% [17] to 59.2% [24]. The level of C18:2 from the southern and central Tunisia populations were 65.30% and 64.76%, respectively (Table 2). Oleic acid was the second most abundant fatty acid identified in *P. harmala* oil, with 23.12%. Values were between 22.28% (for KO) and 24.34% (SE), showing a small difference to previous results [17,24]. Accessions collected from central Tunisia contained more than 24.0% C18:1, except for MH with 22.75%, whereas, in those from southern regions, the C18:1 content was less than 24% and the palmitic acid (C16:0) level was 5.36%. Other studies on *P. harmala* seed oil reported similar palmitic acid contents of 7% [17] and 8.4% [24].

The C18:0 content ranged from a minimum of 2.88% (GF) to a maximum of 3.31% (MD), yielding a mean of 3.08%. Similar mean contents of C18:0 were detected in the southern and central Tunisia accessions (Table 2). A narrow range of C18:0 content (3.0–3.4%), overlapping with the range found in this work, was previously reported [17,24].

#### 2.3.2. Minor Fatty Acids

Linolenic acid (C18:3) was the most abundant minor fatty acid component detected in *P. harmala* seed oils. The minimum and maximum values were 0.21% and 0.84%, respectively, with a mean of 0.65% (Table 2). These values differed from previous reports of 3.6% [17] and 1.4% [24]. Behenic acid (C22:0) was detected in all samples, with a mean content of 0.37%. However, lignoceric acid (C24:0) was detected only in samples from the southern regions, with values of 0.09% (GB), 0.23% (KO) and 0.12% (MD). The remaining minor fatty acids were C18:1 ω11 (0.43%) and C20:0 (0.62%).

The variability in fatty acid contents could be caused by genetic and environmental factors, as mentioned in other studies [25,26,27]. In fact, our samples were obtained from different Tunisian localities with different geographic specificities.

### 2.4. Antifungal Activity

The in vitro antifungal activity of *P. harmala* seed oils against selected fungi is shown in Table 3. The inhibition values of mycelia growth varied from 31.53% (**6**) to 82.11% (**3**) at the ½ concentration. The ANOVA of antifungal activity data showed significant differences between the fungal isolates and the seed oil concentrations (*p* < 0.05). In addition, we noted a significant fungal isolate × oil concentration interaction (*p* < 0.05). The minima and the maxima concentrations of mycelia growth inhibition varied depending on the fungi tested (Table 3). The *P. harmala* seed oils exhibited excellent activity against (**3**) with mycelia growth inhibition ranging within 56–82%, followed by (**8**)–(**10**) with inhibitions of 15–55.7%, 20–54% and 5–58%, respectively. The seed oil showed moderate inhibition against (**6**), with range of 5.19–42%. The evaluated seed oils manifested promising antifungal activities against the tested fungi except for (**5**), which showed no inhibitory effect. Orange essential oil was found to have potential for controlling *A. dauci* and *A. alternata* [28]. The preponderance of linoleic and oleic acids determined by GC-MS analysis of seed oil of the MD population reinforced the antifungal activity of *P. harmala* shown in our study. This is consistent with other studies on the importance of linolenic, linoleic and oleic acids as essential components of seed oil of many plants such as *Pongamia pinnata*, which exhibited a strong antifungal and antibacterial properties [16,29]. Other studies revealed the effectiveness of *Moringa peregrine* against several microorganisms [30].

Other works have highlighted the activity of essential oils on growth of many bacteria species. Essential oils of *Melaleuca* showed strong inhibitory effects against *E. coli*, *P. aeruginosa* and *S. enteric*, with inhibition of about 89% [31]. Biocontrol strategy is still by far the sustainable alternative control against fungal diseases [32]. Seed oil of *P. harmala* exhibited good antioxidant and anti-inflammatory activities [14] and will be considered in further investigations to identify the compounds responsible for the biological activities of the oil. Recently the biological activities, individually, of isomers of vitamin E (α- and γ-T) were reported [33,34]. [35] mentioned that soybean and palm oils were major commercial sources of tocopherols (20% δ-T and 65% γ-T) and tocotrienols, respectively. To our knowledge, the biological activity of δ-T, separately, has not been well studied and should be considered in further work, especially because we found a high δ-T content in our study.

#### Determination of Minimum Inhibitory Concentrations (MICs) and Percentage of Mycelial Growth Inhibition (MGI)

The MICs ranged within 3.9–125.0 µL/mL (dilutions 1/8 to 1/256). The antifungal activity indicated that seed oil inhibited the growth of all tested fungi at all dilutions, except for (**5**), which was resistant against all concentrations (Table 4). Orange essential oil has shown potential to control *A. dauci* and *A. alternata* [27]. Our results show that the bioactivity of the seed oil depended on the fungi isolates. The MIC values were 3.9 µL/mL for six fungal isolates and 15.6, 62.5 and 125.0 µL/mL for (**6**)*,* (**4**) and (**3**), respectively.

## 3. Materials and Methods

### 3.1. Sources of P. harmala Seeds

*P. harmala* fruits were collected from different central and southern Tunisian sites (Table 5). The geographic coordinates corresponding to the location of each fruit sample are also given in Table 5.

### 3.2. Seed Oil Extraction

The mature seeds collected from fruits were dried and then milled in a K/IKA-WERKE M20 grinder. The seed oil was extracted as follows: 80 g of ground seeds were placed in a cellulose paper cone mixed with 250 mL of *n*-hexane using a Soxhlet extraction apparatus for 8 h. The hexane was then removed by rotary evaporation (model R-210, Büchi, Flawil, Switzerland) at 80 °C and finally stored at −20 °C for further analysis. The oil content was expressed as the lipid content percentage on seed powder. All chemicals and reagents used were of analytical grade and were obtained from Merck (Darmstadt, Germany), Sigma-Aldrich (Steinheim, Germany), Acros Organics (Fair Lawn, NJ, USA) and Fisher Scientific Co. (Leicestershire, UK).

### 3.3. Determination of Tocol Content

The tocols present in *P. harmala* seed oils were analyzed by high performance liquid chromatography (HPLC) according to the ISO 9936 standard. The HPLC system was equipped with a Shimadzu LC-20AT pump (Shimadzu, Kyoto, Japan), a Shimadzu RF 20A fluorescence detector (set at excitation and emission wavelengths of 295 and 330 nm, respectively) and a Rheodyne 7725i manual injector (Rohnert Park, CA, USA). The tocols were separated on a normal phase column (Hypersil silica, 15 cm length × 3 mm internal diameter, 3 µm particle size, Thermo Scientific) at ambient temperature and with mobile phase flow level of 0.5 mL/min. The mobile phase was a 99.5:0.5 (*v*/*v*) *n*-hexane/isopropanol mixture. The data were analyzed using the Shimadzu LC solution software Version 2.53. Of seed oil, 0.5 g was diluted with 50 mL of hexane, and 20 µL of this was injected into the column. A mixture of standard tocol isomers (Sigma Chemical Co., St. Louis, MO, USA.) was dissolved in hexane at 2 μg/mL each and used for identification and quantification of the HPLC peaks. The amount of tocols in extracts was calculated as mg tocols/100 g oil.

### 3.4. Determination of Fatty Acid Composition

The fatty acid methyl esters (FAME) composition was determined by converting the oil to fatty acidmethyl esters by addition of 1 mL of n-hexane to 40 mg of oil followed by 200 µL of sodium methoxide (2 M). The mixture is heated in the bath at 50 °C for a few seconds followed by adding 200 µL HCl (2 N). The top layer (1 µL) was injected onto a GC (Agilent 6890N, Santa Clara, CA, USA) equipped with a flame ionization detector (FID) and a polarcapillary column (HP-Innowax polyethylene glycol, 0.25 mm internal diameter, 30 min length and 0.25 µm film in thickness) to obtain individual peaks of fatty acid methyl esters. The detector temperature was 275 °C and the column temperature was 150 °C held for min and increased at the rate of 15 °C/min to 200 °C and the rate of 2 °C/min to 250 °C and held for 4min. The run time was 45 min. The fatty acid methyl esters peaks were identified comparing their retention times with individual standard FAME (approximately 99% pure purchased from Supelco, Bellefonte, PA, USA) and analyzed with the Agilent Technologies Chemstation A09.01 Software. The relative percentage of the fatty acid was calculated on the basis of the peak area of a fatty acid species to the total peak area of all the fatty acids in the oil sample. Fatty acid methyl esters peak identification was confirmed by GC-MS (NIST 2002 database) operating under similar conditions as used for the GC-FID.

### 3.5. Antifungal Activity of Seed Oil

#### 3.5.1. Fungal Isolates

Ten fungal isolates (*Rhizoctonia solani* (**1**); *Macrophomina phaseolina* (**2**); *Pythium* sp.1 (**3**); *Pythium* sp. 2 (**4**); *Alternaria* sp. (**5**); *Colletotrichum* sp. (**6**); *Monosporascus cannonballus* (**7**); *Fusarium solani* f.sp. *cucurbitae* (**8**); *Fusarium oxysporum* f.sp. *melonis* (**9**); and *Fusarium oxysporum* f.sp. *niveum* (**10**)) were identified and conserved in the Laboratory of Phytopathology of the High Institute of Agronomy of Chott-Mariem in Tunisia. Before testing antifungal activity of seed oil, fungal isolates (one for each species) were grown on Potato Dextrose Agar media for 7–10 days.

#### 3.5.2. Effect of Seed Oil on Mycelia Growth

The *P. harmala* seed oil of the MD population was incorporated into melted agar at a desired final concentration and mixed well. Stock solution of 100 mg/mL was prepared by dissolving 5 g of oil with 0.5 mL of dimethyl sulfoxide (DMSO) and diluted up to 50 mL with distilled water. The serial dilution method (1/2^n^) from the prepared stock solution was used to prepare nine different concentrations: D1, 500 µL oil/500 µL DMSO; D2, 250 µL oil/750 µL DMSO; D3, 125 µL oil/875 µL DMSO; D4, 62.5 µL oil/937.5 µL DMSO; D5, 31.25 µL oil/968.75 µL DMSO; D6, 15.62 µL oil/984.38 µL DMSO; D7, 7.81 µL oil/992.19 µL DMSO; D8, 3.9 µL oil/996.1 µL DMSO; and D9, 1.95 µL oil/998.05 µL DMSO. The negative control was DMSO (1%). Mycelia disks (0.7-mm diameter), from five-day-old cultures, of the isolates used were deposited in the center of the plates. Mycelium growth was measured after 48, 72 and 96 h of incubation in darkness at 25 °C. Three replicates were used for each concentration. Each measure was compared with the control cultures under the same conditions. The percent inhibition of growth diameter (IP) was calculated accordingly to the following formula:

IP = (C − T)/C × 100

where C and T represent the mean diameter of three replicates of mycelia growth in control and treated slides, respectively.

### 3.6. Statistical Analysis

A one-way analysis of variance (ANOVA) procedure was applied, using Minitab Statistical Software Release 14 (Minitab Inc., State College, PA, USA). Significance differences between reported means were established at *p* < 0.05 according to Tukey’s test.

## 4. Conclusions

All seed oils of *P. harmala* populations contained very high levels of tocols (2722.68 mg/100 g), with δ-T the most abundant isomer (2441.78 mg/100 g) followed by γ-T (205.46 mg/100 g). We noted also the presence of tocotrienols for the first time in *P. harmala*. The preponderance of the two components (δ- and γ-T) explained the pertinent antifungal activity against the selected fungi and suggests that *P. harmala* has promise as an alternative approach for biological control of other microorganisms. In addition, the high levels in fatty acids in the *P. harmala* seeds, compared with other plants cited in the literature, revealed the importance of this plant as a new potential source of biofuel and for further medicinal applications. This idea is supported by the wide geographic spread of *P. harmala* in Tunisia and the adaptability of this species to various climactic conditions.

## Figures and Tables

**Table 1:** Means ± sd of tocol contents as (mg/100 g and percent) in *P. harmala* seed oil samples.

Population	Tocopherols								Tocotrienols						Tocols
	α-T		β-T		γ-T		δ-T		α-T3		γ-T3		δ-T3		
	Contents														Total
**GF**	51.05 * ± 1.51 ^a^	1.78 ± 0.05 ^a^	9.23 ± 0.17 ^a,b^	0.31 ± 0.01 ^a,b^	213.86 ± 3.45 ^a^	7.45 ± 0.12 ^a,b,c^	2573.03 ± 2.70 ^a^	89.63 ± 0.06 ^a^	3.92 ± 0.08 ^a^	0.14 ± 0.01 ^a^	2.97 ± 0.01	0.10 ± 0.00	16.87 ± 0.14 ^a^	0.59 ± 0.01^a^	2870.98 ± 0.95 ^a^
**GB**	29.96 ± 0.85 ^b^	1.14 ± 0.04 ^b^	9.91 ± 0.72 ^a,c^	0.37 ± 0.03 ^c,d^	211.57 ± 2.14 ^a^	8.33 ± 0.50 ^a^	2366.17 ± 4.12 ^b^	89.72 ± 0.04 ^a,b^	2.78 ± 0.08 ^b^	0.11 ± 0.01 ^b^	-	-	17.03 ± 0.58 ^a^	0.65 ± 0.02^a^	2637.43 ± 5.47 ^b^
**KO**	17.77 ± 1.56 ^c^	0.86 ± 0.03 ^c,d^	6.17 ± 0.11 ^d^	0.28 ± 0.01^b^	176.77 ± 1.77 ^b^	7.89 ± 0.08 ^a,b^	2022.42 ± 3.32 ^c^	90.30 ± 0.08 ^c^	1.68 ± 0.06 ^c^	0.08 ± 0.01 ^c^	-	-	13.47 ± 0.38 ^b^	0.60 ± 0.01^a^	2239.77 ± 1.91 ^c^
**MD**	30.03 ± 0.69 ^b^	0.80 ± 0.02 ^d^	10.80 ± 0.24 ^c^	0.29 ± 0.01^ab^	277.62 ± 3.54 ^c^	7.36 ± 0.13 ^b,c^	3424.41 ± 17.42 ^d^	90.81 ± 0.10 ^d^	3.82 ± 0.07 ^a^	0.10 ± 0.00 ^b^	-	-	24.30 ± 0.30 ^c^	0.65 ± 0.01^a^	3770.98 ± 15.19 ^d^
**MH**	23.37 ± 0.62 ^d^	0.89 ± 0.03 ^c,d^	8.81 ± 0.10 ^a,b^	0.34 ± 0.01 ^a,d^	209.76 ± 2.05 ^a^	7.99 ± 0.06 ^a,b^	2363.68 ± 3.35 ^b^	90.06 ± 0.03 ^b,c^	2.33 ± 0.06 ^d^	0.09 ± 0.00 ^b,c^	-	-	16.12 ± 0.96 ^a^	0.62 ± 0.04^a^	2624.57 ± 4.51 ^b^
**SE**	20.36 ± 0.50 ^c,d^	0.97 ± 0.01 ^c^	8.50 ± 0.24 ^b^	0.40 ± 0.01 ^c^	143.18 ± 2.63 ^d^	6.84 ± 0.16 ^c^	1900.51 ± 10.99 ^e^	90.81 ± 0.17 ^d^	4.27 ± 0.11 ^e^	0.21 ± 0.01 ^d^	-	-	16.00 ± 0.54 ^a^	0.76 ± 0.03^b^	2092.81 ± 8.19 ^e^

* Means within the same column with different superscript letters (a, b, c, d, e) are significantly different (*p* < 0.05). GF, Gafsa; GB, Gabes; KO, Koutine; MD, Mednine; SE, Sousse; MH, Mahdia.

**Table 2:** Means ± sd of major and minor fatty acids detected in seed oil samples of six *P. harmala* populations.

Population	C16:0	C18:0	C18:1ω7	C18:1ω9	C18:2	C18:1ω11	C18:3 ω3	C20:0	C22:0	C24:0
**GF**	5.00 ± 0.03 ^a^	2.88 * ± 0.03 ^a^	1.12 ± 0.02 ^a,b^	23.42 ± 0.07 ^a^	65.90 ± 0.08 ^a^	0.37 ± 0.03 ^a^	0.21 ± 0.00 ^a^	0.74 ± 0.04 ^a,b^	0.32 ± 0.03 ^a^	-
**GB**	5.54 ± 0.04 ^b^	2.97 ± 0.00 ^a^	1.19 ± 0.01 ^a,c^	22.86 ± 0.08 ^b,c^	65.65 ± 0.01 ^b^	0.23 ± 0.00 ^b^	0.63 ± 0.01 ^b^	0.65 ± 0.04 ^b,c^	0.20 ± 0.00 ^b^	0.09 ± 0.03 ^a^
**KO**	5.49 ± 0.04 ^b^	3.12 ± 0.00 ^b^	1.04 ± 0.01 ^b^	22.28 ± 0.11 ^d^	65.30 ± 0.07 ^c^	0.51 ± 0.01 ^c^	0.84 ± 0.03 ^c^	0.60 ± 0.00 ^c^	0.57 ± 0.00 ^c^	0.23 ± 0.00 ^b^
**MD**	5.46 ± 0.03 ^b^	3.31 ± 0.03 ^c^	1.08 ± 0.01 ^a,b^	23.05 ± 0.04 ^b^	64.36 ± 0.04 ^d^	0.59 ± 0.00 ^d^	0.81 ± 0.04 ^c^	0.78 ± 0.00 ^a^	0.38 ± 0.01 ^a,d^	0.12 ± 0.00 ^a^
**SE**	5.51 ± 0.06 ^b^	2.97 ± 0.01 ^a^	1.19 ± 0.03 ^a,c^	24.34 ± 0.00 ^e^	64.76 ± 0.06 ^e^	-	0.58 ± 0.03 ^b^	0.26 ± 0.00 ^d^	0.36 ± 0.01 ^a,d^	-
**MH**	5.16 ± 0.07 ^a^	3.25 ± 0.04 ^c^	1.29 ± 0.06 ^c^	22.75 ± 0.07 ^c^	65.09 ± 0.01 ^c^	0.52 ± 0.03 ^c^	0.82 ± 0.06 ^c^	0.69 ± 0.00 ^a,b,c^	0.40 ± 0.01 ^d^	-

* Means within the same column with different superscript letters (a, b, c, d, e) are significantly different (*p* < 0.05). GF, Gafsa; GB, Gabes; KO, Koutine; MD, Medenine; SE, Sousse; MH, Mahdia.

**Table 3:** Antifungal activity (%) of different seeds oil concentrations of *P. harmala* on 10 fungal isolates.

Fungal Isolates	Seeds Oil Concentrations									
	1/2	1/4	1/8	1/16	1/32	1/64	1/128	1/256	1/512	DMSO
**1**	33.46	30.76	28.84	21.61	21.15	23.84	16.15	15.38	-	-
**2**	36.53	37.30	37.30	31.73	29.42	15.38	9.61	5.76	-	-
**3**	82.11	76.92	56.34	-	-	-	-	-	-	-
**4**	42.11	41.73	30.76	30.19	5.61	5.19	-	-	-	-
**5**	-	-	-	-	-	-	-	-	-	-
**6**	31.53	31.34	14.80	15.38	-	-	-	-	-	-
**7**	53.84	51.92	35.38	37.80	38.46	28.84	20.57	20.00	-	-
**8**	62.88	47.50	35.19	29.42	35.19	41.15	37.30	36.53	-	-
**9**	55.70	29.61	26.34	25.76	11.53	20.57	15.96	14.86	-	-
**10**	57.69	53.84	39.23	26.15	21.15	22.88	5.76	5.19	-	

*Rhizoctonia solani* (**1**); *Macrophomina phaseolina* (**2**); *Pythium* sp.1 (**3**); *Pythium* sp. 2 (**4**); *Alternaria* sp. (**5**); *Colletotrichum* sp. (**6**); *Monosporascus cannonballus* (**7**); *Fusarium solani* f.sp. *cucurbitae* (**8**); *Fusarium oxysporum* f.sp. *melonis* (**9**); and *Fusarium oxysporum* f.sp. *niveum* (**10**).

**Table 4:** Percentages of mycelial growth inhibition (MGI) and the minimum inhibitory concentration (CMI) of *P. harmala* seeds oil on 10 fungal pathogen species.

Pathogens	MGI (%)	CMI (µL/mL)
**1**	33.4	3.9
**2**	31.75	3.9
**3**	82.11	125
**4**	41.73	15.62
**5**	0.00	-
**6**	31.53	62.5
**7**	51.92	3.9
**8**	62.88	3.9
**9**	55.76	3.9
**10**	57.69	3.9

*Rhizoctonia solani* (**1)**; *Macrophomina phaseolina* (**2**); *Pythium* sp.1 (**3**); *Pythium* sp. 2 (**4**); *Alternaria* sp. (**5**); *Colletotrichum* sp. (**6**); *Monosporascus cannonballus* (**7**); *Fusarium solani* f.sp. *cucurbitae* (**8**); *Fusarium oxysporum* f.sp. *melonis* (**9**); and *Fusarium oxysporum* f.sp. *niveum* (**10**).

**Table 5:** Geographical coordinates corresponding to the location of collected *P. harmala* fruits samples and oil seed yields.

Code	Region Name	Location	Latitude	Longitude	Altitude (m)	Oil Content (%)
GF	Gafsa	South Tunisia	34 25 N	08 47 E	298	5.72
GB	Gabes	South Tunisia	33 52 N	10 05 E	9	13.54
KO	Koutine	South Tunisia	33 45 N	10 34 E	81	14.75
MD	Mednine	South Tunisia	33 21 N	10 30 E	90	13.83
MH	Mahdia	Central Tunisia	35 30 N	11 03 E	7	13.98
SE	Sousse	Central Tunisia	35 49 N	10 38 E	24	13.72

## Supplementary Materials

The following are available online.
