# Supplementary material for: High Reserve in δ-Tocopherol of Peganum harmala Seeds Oil and Antifungal Activity of Oil against Ten Plant Pathogenic Fungi
Source: Molecules. 2020 Oct 6;25(19):4569. doi: 10.3390/molecules25194569 (PMC7582963; doi:10.3390/molecules25194569)
Supplement: Supplementary file 1 [file molecules-25-04569-s001.pdf]

KING SAUD UNIVERSITY
COLLEGE OF SCIENCE / CHEMISTRY DEPARTMENT

Sample Information

Acquired by	: Dr. Nehdi I. A
Sample Name	: GAB Peganum harmala 0.02g 25 2012 2 3 16
Sample ID	: GAB Peganum harmala 0.02g 25 20
Injection Volume	: 25 uL
Data Filename	: GAB Peganum harmala 0.02g 25 2012 2 3 16.lcd
Method Filename	: tocopherols analysis hex isoprop 99.5 0.5.lcm
R[illegible]	[illegible]
D[illegible]	
D[illegible]	

Chromatogram

GAB Peganum harmala 0.02g 25 2012 2 3 16 C:\...\LCsolution\data files imed\GAB Peganum harmala 0.02g 25 2012 2 3 16.lcd

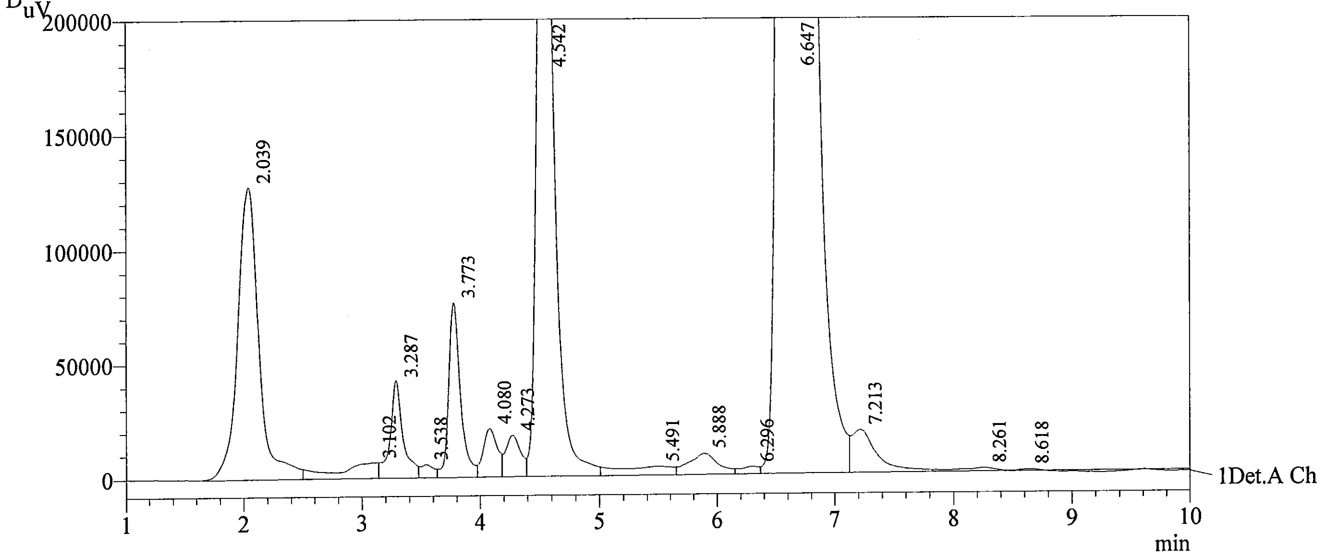

1 Det.A Ch1 / 295nm - 330nm

PeakTable

Detector A Ch1 295nm - 330nm

Peak#	Name	Ret. Time	Height	Area	Area %
1		2.039	127568	1589355	3.523
2		3.102	6739	163334	0.362
3		3.287	42789	322016	0.714
4		3.538	5607	45014	0.100
5		3.773	76344	534964	1.186
6		4.080	21053	173199	0.384
7		4.273	17928	160590	0.356
8		4.542	407010	3428007	7.598
9		5.491	3633	120548	0.267
10		5.888	9122	156704	0.347
11		6.296	3094	33980	0.075
12		6.647	3533658	38333806	84.968
13		7.213	18727	275887	0.612
14		8.261	1348	29655	0.066
15		8.618	612	-251440	-0.557
Total			4275231	45115617	100.000

Sample Information

Analyzed by : Admin
Analyzed : 2/1/2016 6:18:42 PM
Sample Type : Unknown
Level # : 1
Sample Name : GAB hajji
Sample ID :
Vial # : 6
Injection Volume : 1.00
Data File : C:\GCMSsolution\Data\Project1\FAME\GAB hajji.qgd
Method File : C:\GCMSsolution\Data\Project1\FAME\FAME-1,5 F O115 R 2-.qgm
Report File :

Top Position

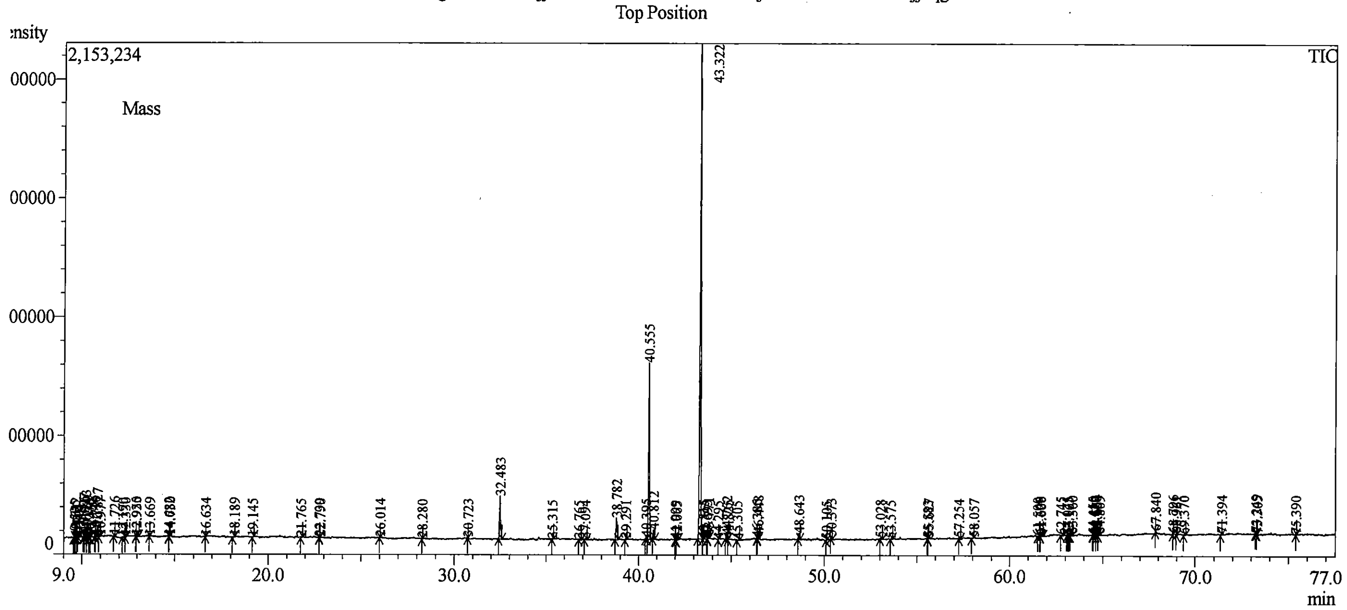

Peak Report TIC

Peak#	R.Time	Name	Base m/z	Area	Area%
1	9.595		90.95	73537	0.50
2	9.632		90.95	101337	0.69
3	9.715		470.95	2707	0.02
4	9.801		57.95	24260	0.16
5	10.097		90.95	54643	0.37
6	10.203		105.00	104744	0.71
7	10.260		104.95	35729	0.24
8	10.370		470.95	11648	0.08
9	10.452		471.85	5732	0.04
10	10.710		105.00	18971	0.13
11	10.827		105.00	114287	0.77
12	10.977		119.00	9314	0.06
13	11.726		105.00	16544	0.11
14	12.190		471.85	25180	0.17
15	12.330		471.85	11734	0.08
16	12.925		471.85	3590	0.02
17	12.950		471.80	20412	0.14
18	13.669		471.80	3856	0.03
19	14.682		471.80	6388	0.04
20	14.730		471.80	7771	0.05
21	16.634		471.85	4788	0.03
22	18.189		471.85	22407	0.15
23	19.145		471.85	8692	0.06
24	21.765		471.85	14762	0.10
25	22.739		471.80	6939	0.05
26	22.790		471.85	7631	0.05
27	26.014		471.85	4403	0.03
28	28.280		471.80	7508	0.05
29	30.723		471.80	4449	0.03
30	32.483	C16	74.00	741738	5.01
31	35.315		471.80	12292	0.08
32	36.765		471.85	21944	0.15
33	37.094		471.85	27213	0.18
34	38.782	C18	74.00	385451	2.61
35	39.291		472.85	27394	0.19
36	40.395		74.00	30625	0.21
37	40.555	C18/1 w9	54.95	3034608	20.52
38	40.812	C18/1 w7	54.95	158558	1.07
39	41.999		471.85	4836	0.03
40	42.065		472.85	26492	0.18
41	43.322	C18/2 w6	67.00	8714487	58.91
42	43.515		471.80	11965	0.08
43	43.695		471.80	7831	0.05
44	43.771	Apid	221.90	51172	0.35
45	44.295		471.80	2998	0.02

C40 H82 tetracontane

Peak#	R.Time	Name	Base m/z	Area	Area%
46	44.762	C 20	74.00	86568	0.59
47	44.825		471.80	5316	0.04
48	45.305		471.80	25614	0.17
49	46.385		471.80	16896	0.11
50	46.448	C 18/3 w3	79.00	83828	0.57
51	48.643	C40H82 Tetra	57.00	89101	0.60
52	50.105		471.80	7188	0.05
53	50.373	C 22	73.95	26599	0.18
54	53.028		69.00	28162	0.19
55	53.575		473.80	8932	0.06
56	55.583		471.80	5211	0.04
57	55.627		471.80	11988	0.08
58	57.254		473.85	30478	0.21
59	58.057		471.85	21037	0.14
60	61.500		471.80	10001	0.07
61	61.608		471.80	9875	0.07
62	61.660		471.80	33498	0.23
63	62.745		471.80	28872	0.20
64	63.075		472.80	15719	0.11
65	63.165		471.85	14000	0.09
66	63.360		471.80	34547	0.23
67	64.455		471.85	7962	0.05
68	64.510		471.80	23307	0.16
69	64.600		471.80	8135	0.05
70	64.654		54.95	44966	0.30
71	64.809		471.80	30283	0.20
72	67.840		471.80	3240	0.02
73	68.796		472.85	4874	0.03
74	68.961		473.80	9023	0.06
75	69.370		471.80	23492	0.16
76	71.394		471.80	22503	0.15
77	73.249		471.80	14265	0.10
78	73.305		471.80	5529	0.04
79	75.390		471.80	7155	0.05
				14791731	100.00

KING SAUD UNIVERSITY
COLLEGE OF SCIENCE / CHEMISTRY DEPARTMENT

Sample Information

Acquired by	: Dr. Nehdi I. A
Sample Name	: GF 0.02g 25 2012 2 3 16
Sample ID	: GF 0.02g 25 2012 2 3 16
Injection Volume	: 25 uL
Data Filename	: GF 0.02g 25 2012 2 3 16.lcd
Method Filename	: tocopherols analysis hex isoprop 99.5 0.5.lcm
R[illegible]	: [illegible]
D	
D	

Chromatogram

GF 0.02g 25 2012 2 3 16 C:\LabSolutions\LCsolution\data files imed\GF 0.02g 25 2012 2 3 16.lcd

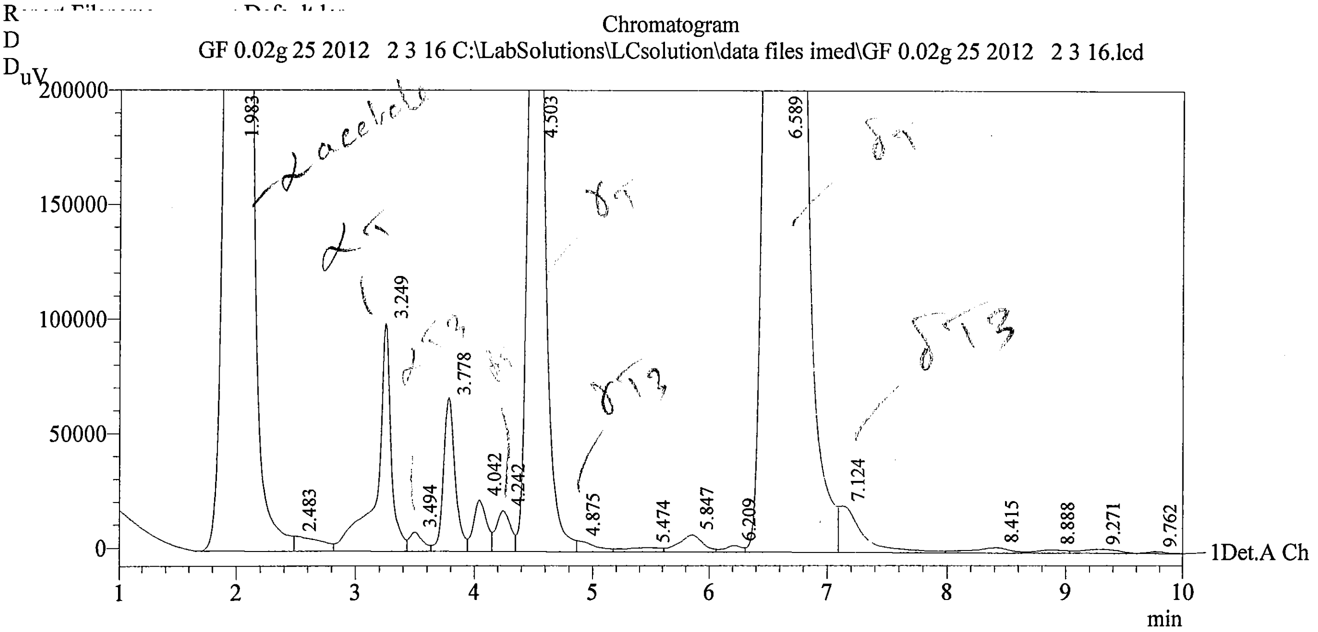

1 Det.A Ch1 / 295nm - 330nm

PeakTable

Detector A Ch1 295nm - 330nm

Peak#	Name	Ret. Time	Height	Area	Area %
1		1.983	1468116	14048236	22.817
2		2.483	6410	97457	0.158
3		3.249	99507	827090	1.343
4		3.494	8261	63589	0.103
5		3.778	67180	458918	0.745
6		4.042	22107	173988	0.283
7		4.242	17510	149475	0.243
8		4.503	420063	3464825	5.628
9		4.875	4664	48090	0.078
10		5.474	1743	37312	0.061
11		5.847	7379	103790	0.169
12		6.209	2733	28813	0.047
13		6.589	3819372	41685110	67.704
14		7.124	20242	273293	0.444
15		8.415	2152	46888	0.076
16		8.888	1359	22461	0.036
17		9.271	1664	35699	0.058
18		9.762	659	4172	0.007
Total			5971122	61569207	100.000

Peak#	R.Time	Name	Base m/z	Area	Area%
46	74.099		471.90	23232	0.28
				8382012	100.00

Sample Information

Analyzed by : Admin
Analyzed : 2/1/2016 12:53:30 PM
Sample Type : Unknown
Level # : 1
Sample Name : GF hajji
Sample ID :
Vial # : 2
Injection Volume : 1.00
Data File : C:\GCMSsolution\Data\Project1\FAME\GF hajji.qgd
Method File : C:\GCMSsolution\Data\Project1\FAME\FAME-1,5 F O115 R 2-.qgm
Report File :

Chromatogram GF hajji C:\GCMSsolution\Data\Project1\FAME\GF hajji.qgd
Top Position

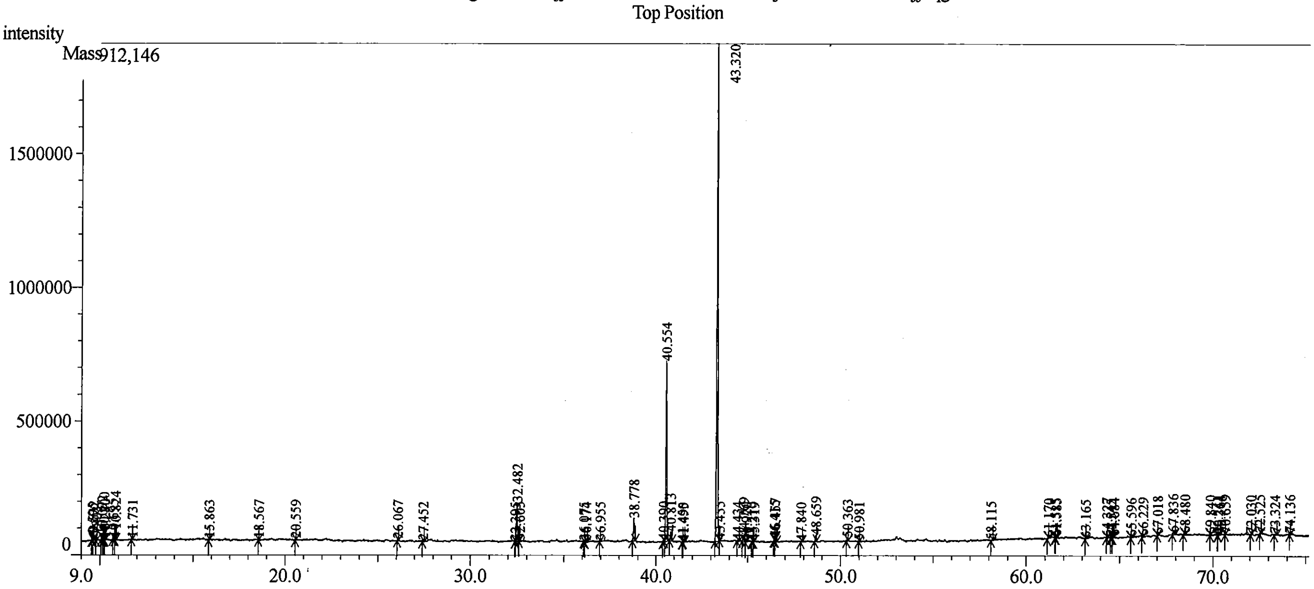

Peak Report TIC

Peak#	R.Time	Name	Base m/z	Area	Area%
1	9.595		91.00	55324	0.43
2	9.632		91.00	67787	0.52
3	9.803		58.00	20802	0.16
4	10.092		91.00	59105	0.46
5	10.200		105.00	87825	0.68
6	10.270		105.00	23264	0.18
7	10.695		105.05	21235	0.16
8	10.824		105.00	110104	0.85
9	11.731		105.00	16044	0.12
10	15.863		472.05	16204	0.13
11	18.567		472.05	6974	0.05
12	20.559		473.05	6779	0.05
13	26.067		472.05	21169	0.16
14	27.452		472.05	23035	0.18
15	32.395		471.15	5286	0.04
16	32.482		74.00	589538	4.56
17	32.605		472.05	5971	0.05
18	36.075		474.05	11560	0.09
19	36.174		472.05	27051	0.21
20	36.955		472.05	9707	0.08
21	38.778		74.00	339947	2.63
22	40.390		472.05	43670	0.34
23	40.554		55.00	2759790	21.34
24	40.813		55.00	133597	1.03
25	41.450		473.95	10307	0.08
26	41.495		472.05	22693	0.18
27	43.320		67.00	7767111	60.07
28	43.455		472.05	3003	0.02
29	44.434		472.05	12763	0.10
30	44.769		74.00	87191	0.67
31	44.920		472.05	17646	0.14
32	45.210		472.00	18299	0.14
33	45.319		472.05	7645	0.06
34	46.415		83.10	25285	0.20
35	46.457		472.05	45877	0.35
36	47.840		472.00	13487	0.10
37	48.659		57.00	79671	0.62
38	50.363		74.00	38833	0.30
39	50.981		472.00	7624	0.06
40	58.115		472.05	9191	0.07
41	61.170		472.00	15847	0.12
42	61.515		473.05	5710	0.04
43	61.585		474.05	24456	0.19
44	63.165		472.05	6554	0.05
45	64.327		472.00	7285	0.06

GF

Peak#	R.Time	Name	Base m/z	Area	Area%
46	64.545		474.05	7424	0.06
47	64.684		472.00	24666	0.19
48	65.596		472.00	4686	0.04
49	66.229		472.05	15892	0.12
50	67.018		472.00	4433	0.03
51	67.836		472.00	60044	0.46
52	68.480		278.95	25856	0.20
53	69.840		472.00	4355	0.03
54	70.241		472.05	6115	0.05
55	70.364		473.05	16326	0.13
56	70.659		473.05	3652	0.03
57	72.030		473.00	5681	0.04
58	72.525		472.05	13686	0.11
59	73.324		472.00	23560	0.18
60	74.136		472.00	8116	0.06
61	75.779		472.00	13437	0.10
62	76.331		472.00	4683	0.04
				12930858	100.00

KING SAUD UNIVERSITY
COLLEGE OF SCIENCE / CHEMISTRY DEPARTMENT

Sample Information

Acquired by	: Dr. Nehdi I. A
Sample Name	: KOU Peganum harmala 0.02g 25 2012 2 3 16
Sample ID	: KOU Peganum harmala 0.02g 25 20
Injection Volume	: 25 uL
Data Filename	: KOU Peganum harmala 0.02g 25 2012 2 3 16.lcd
Method Filename	: tocopherols analysis hex isoprop 99.5 0.5.lcm
R[illegible]	: [illegible]

Chromatogram

D KOU Peganum harmala 0.02g 25 2012 2 3 16 C:\...\LCsolution\data files imed\KOU Peganum harmala 0.02g 25 2012 2 3 16.lcd

D

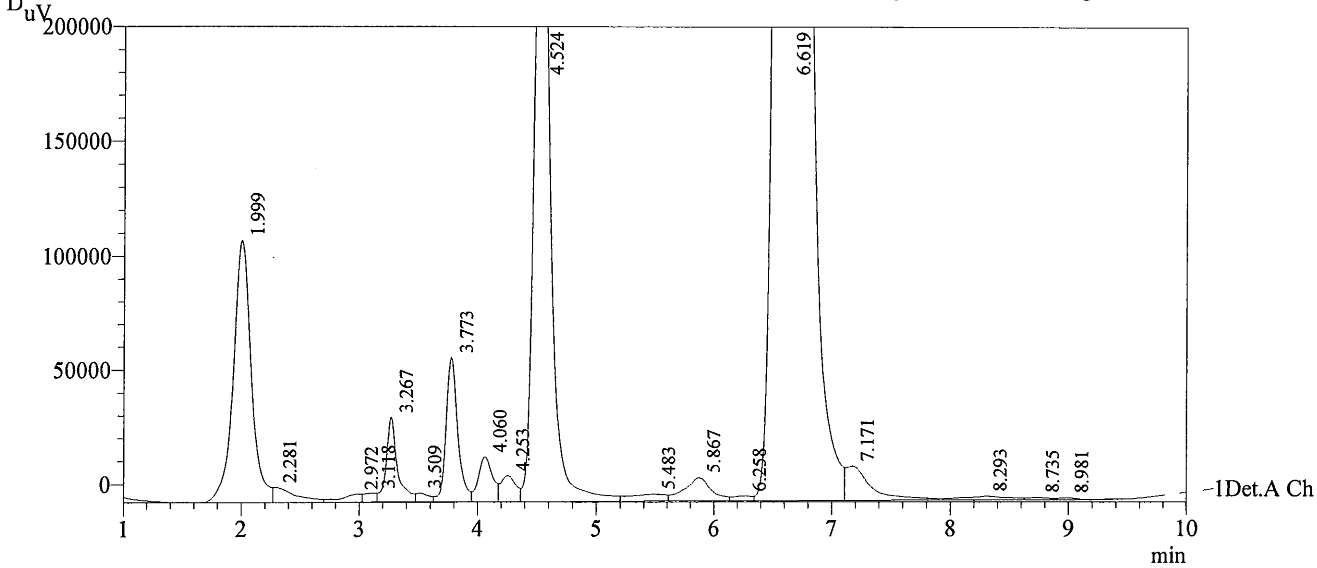

1 Det.A Ch1 / 295nm - 330nm

PeakTable

Detector A Ch1 295nm - 330nm

Peak#	Name	Ret. Time	Height	Area	Area %
1		1.999	114732	1207880	3.140
2		2.281	6512	85264	0.222
3		2.972	3320	40259	0.105
4		3.118	3807	27458	0.071
5		3.267	36881	244429	0.635
6		3.509	3722	27310	0.071
7		3.773	62870	430836	1.120
8		4.060	19342	161456	0.420
9		4.253	11241	99995	0.260
10		4.524	337127	2863864	7.445
11		5.483	2825	60272	0.157
12		5.867	10025	156502	0.407
13		6.258	1935	21579	0.056
14		6.619	3022153	32764914	85.179
15		7.171	14887	218075	0.567
16		8.293	1515	39707	0.103
17		8.735	904	10607	0.028
18		8.981	647	5654	0.015
Total			3654444	38466060	100.000

Sample Information

Analyzed by	: Admin
Analyzed	: 2/1/2016 2:14:43 PM
Sample Type	: Unknown
Level #	: 1
Sample Name	: KOU hajji
Sample ID	:
Vial #	: 3
Injection Volume	: 1.00
Data File	: C:\GCMSsolution\Data\Project1\FAME\KOU hajji.qgd
Method File	: C:\GCMSsolution\Data\Project1\FAME\FAME-1,5 F O115 R 2-.qgm
Report File	:

Chromatogram KOU hajji C:\GCMSsolution\Data\Project1\FAME\KOU hajji.qgd
Top Position

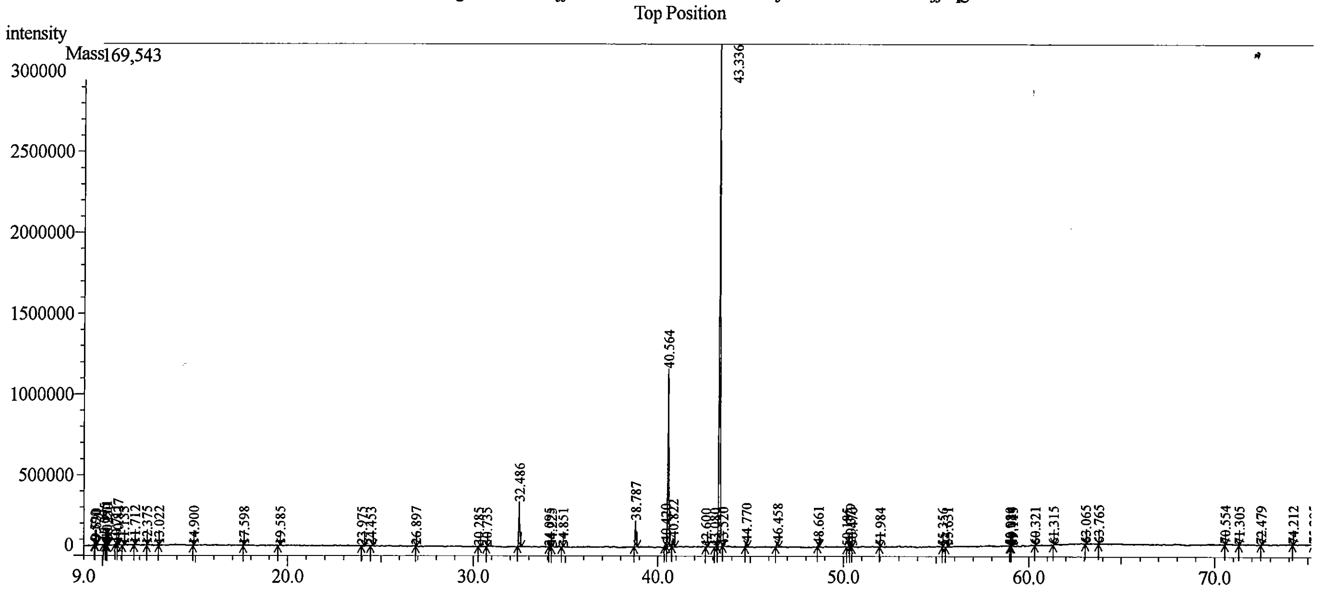

Peak Report TIC

Peak#	R.Time	Name	Base m/z	Area	Area%
1	9.590		91.00	35615	0.17
2	9.630		91.00	53312	0.25
3	10.095		91.00	52638	0.25
4	10.201		105.05	104889	0.49
5	10.250		105.00	37939	0.18
6	10.711		105.05	22957	0.11
7	10.827		105.05	120593	0.57
8	11.133		471.95	22155	0.10
9	11.712		105.00	18425	0.09
10	12.375		471.95	5247	0.02
11	13.022		471.95	5139	0.02
12	14.900		472.00	9531	0.04
13	17.598		472.00	12115	0.06
14	19.585		471.95	20406	0.10
15	23.975		472.00	33326	0.16
16	24.453		471.95	26456	0.12
17	26.897		472.00	9812	0.05
18	30.285		471.95	20988	0.10
19	30.735		472.00	11138	0.05
20	32.486		74.00	1115307	5.25
21	34.095		471.95	23029	0.11
22	34.225		471.95	3211	0.02
23	34.851		471.95	15454	0.07
24	38.787		74.00	633259	2.98
25	40.420		74.00	103651	0.49
26	40.564		54.95	4522911	21.30
27	40.822		55.00	210606	0.99
28	42.600		471.95	8935	0.04
29	43.080		471.95	25424	0.12
30	43.336		67.00	13252291	62.42
31	43.520		471.95	3875	0.02
32	44.770		74.00	122237	0.58
33	46.458		79.00	170139	0.80
34	48.661		57.00	37477	0.18
35	50.185		471.95	29179	0.14
36	50.379		74.00	117025	0.55
37	50.470		471.95	8326	0.04
38	51.984		472.00	8583	0.04
39	55.356		471.95	5235	0.02
40	55.631	C24 lignoceric	74.00	46967	0.22
41	58.980		471.95	5233	0.02
42	59.024		471.95	10482	0.05
43	59.115		471.95	12149	0.06
44	60.321		471.95	13466	0.06
45	61.315		167.60	3426	0.02

C18:1 - ω11

Ligonoceric

Peak#	R.Time	Name	Base m/z	Area	Area%
46	63.065		471.95	8933	0.04
47	63.765		471.95	7149	0.03
48	70.554		471.95	6089	0.03
49	71.305		471.95	5905	0.03
50	72.479		471.95	16394	0.08
51	74.212		471.05	10216	0.05
52	75.305		471.95	8510	0.04
53	76.389		471.95	20705	0.10
54	76.918		471.95	5172	0.02
55	77.125		472.95	12355	0.06
				21231986	100.00

[illegible] il [illegible]

48,64 i [illegible]

KING SAUD UNIVERSITY
COLLEGE OF SCIENCE / CHEMISTRY DEPARTMENT

Sample Information

Acquired by	: Dr. Nehdi I. A
Sample Name	: Med oil 0.02g 25 2012 2 3 16
Sample ID	: Med oil 0.02g 25 2012 2 3
Injection Volume	: 25 uL
Data Filename	: Med oil citrillus oil 0.02g 25 2012 2 3 16.lcd
Method Filename	: tocopherols analysis hex isoprop 99.5 0.5.lcm
R[illegible]	[illegible]
D	
D	

Chromatogram

Med oil 0.02g 25 2012 2 3 16 C:\LabSolutions\LCsolution\data files imed\Med oil citrillus oil 0.02g 25 2012 2 3 16.lcd

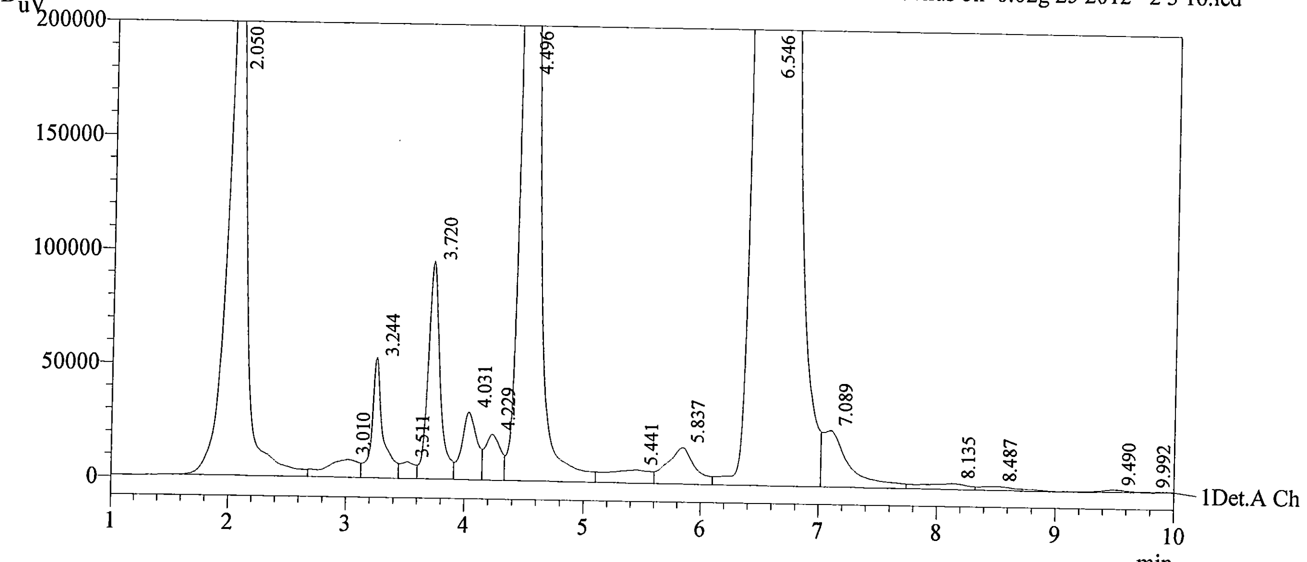

1 Det.A Ch1 / 295nm - 330nm

PeakTable

Detector A Ch1 295nm - 330nm

Peak#	Name	Ret. Time	Height	Area	Area %
1		2.050	246043	2643453	4.054
2		3.010	7627	146977	0.225
3		3.244	52697	339531	0.521
4		3.511	7157	61845	0.095
5		3.720	95484	667262	1.023
6		4.031	29453	265118	0.407
7		4.229	19942	175021	0.268
8		4.496	533705	4497621	6.898
9		5.441	5610	149003	0.229
10		5.837	15882	263844	0.405
11		6.546	5193324	55478217	85.087
12		7.089	24647	393756	0.604
13		8.135	2572	70465	0.108
14		8.487	1702	37578	0.058
15		9.490	919	12218	0.019
16		9.992	-18	-312	-0.000
Total			6236745	65201597	100.000

Sample Information

Analyzed by	: Admin
Analyzed	: 2/1/2016 11:32:08 AM
Sample Type	: Unknown
Level #	: 1
Sample Name	: MED hajji
Sample ID	:
Vial #	: 1
Injection Volume	: 1.00
Data File	: C:\GCMSsolution\Data\Project1\FAME\MED hajji.qgd
Method File	: C:\GCMSsolution\Data\Project1\FAME\FAME-1,5 F O115 R 2-.qgm
Report File	:

Top Position

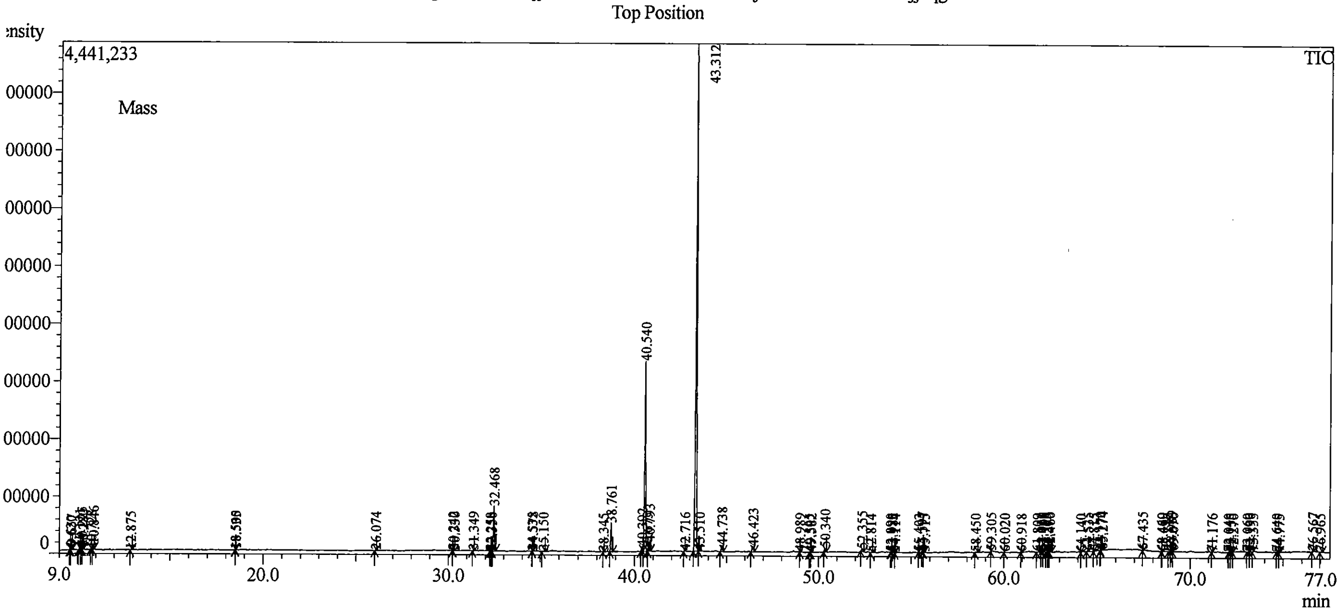

Peak Report TIC

Peak#	R.Time	Name	Base m/z	Area	Area%
1	9.620		91.00	65813	0.21
2	9.657		91.05	78571	0.25
3	10.121		91.00	59558	0.19
4	10.225		105.05	85346	0.27
5	10.280		120.10	35190	0.11
6	10.728		105.05	13853	0.04
7	10.846		105.00	110143	0.35
8	12.875		472.15	27532	0.09
9	18.500		473.10	4466	0.01
10	18.535		472.10	2134	0.01
11	26.074		74.00	26405	0.08
12	30.242		472.10	9928	0.03
13	30.330		472.10	12322	0.04
14	31.349		472.10	26076	0.08
15	32.258		472.10	14682	0.05
16	32.350		472.10	12638	0.04
17	32.468	C16	74.00	1615979	5.14
18	34.535		474.05	12645	0.04
19	34.578		473.10	27587	0.09
20	35.150		472.10	36799	0.12
21	38.345		473.10	9114	0.03
22	38.761	C18	74.00	988263	3.14
23	40.392		55.00	177633	0.57
24	40.540	C18/1 w9	55.00	6872412	21.86
25	40.675		472.10	3881	0.01
26	40.793	C18/2 w'7	55.00	322145	1.02
27	42.716		472.10	21291	0.07
28	43.312	C18/2 w6	67.00	19198901	61.08
29	43.510	Apiol	472.10	17245	0.05
30	44.738	C20	74.00	232853	0.74
31	46.423	C18/3	79.00	243755	0.78
32	48.989	C40 H82	472.10	4584	0.01
33	49.505		472.10	8962	0.03
34	49.582		472.10	7101	0.02
35	50.340		74.00	142933	0.45
36	52.355	C22	149.00	113267	0.36
37	52.814		472.05	21273	0.07
38	53.890		472.05	6332	0.02
39	53.985		473.05	46087	0.15
40	54.114		475.15	16294	0.05
41	55.403		473.10	6920	0.02
42	55.597		74.00	36864	0.12
43	55.715		53.95	14211	0.05
44	58.450		473.05	27775	0.09
45	59.305		473.10	3735	0.01

Peak#	R.Time	Name	Base m/z	Area	Area%
46	60.020		472.05	4784	0.02
47	60.918		472.05	8374	0.03
48	61.809		472.10	21700	0.07
49	62.010		472.10	26546	0.08
50	62.106		472.10	19840	0.06
51	62.265		474.10	23329	0.07
52	62.370		473.10	41161	0.13
53	62.460		472.10	51416	0.16
54	64.140		472.05	3060	0.01
55	64.535		472.05	19244	0.06
56	64.815		472.05	1969	0.01
57	65.170		472.10	7347	0.02
58	65.274		472.05	22203	0.07
59	67.435		472.10	7238	0.02
60	68.460		472.05	5627	0.02
61	68.489		472.10	7537	0.02
62	68.810		472.05	44992	0.14
63	68.989		267.25	116520	0.37
64	69.070		172.00	9101	0.03
65	71.176		472.10	7520	0.02
66	72.040		473.05	23564	0.07
67	72.155		472.10	6225	0.02
68	72.270		472.10	3551	0.01
69	73.060		472.10	19972	0.06
70	73.190		472.05	5697	0.02
71	73.355		472.05	7439	0.02
72	74.640		472.10	27452	0.09
73	74.775		472.10	15307	0.05
74	76.567		472.05	26818	0.09
75	76.965		472.10	26573	0.08
				31433604	100.00

KING SAUD UNIVERSITY
COLLEGE OF SCIENCE / CHEMISTRY DEPARTMENT

Sample Information

Acquired by	: Dr. Nehdi I. A
Sample Name	: MAH Peganum harmala 0.02g 25 2012 2 3 16
Sample ID	: MAH Peganum harmala 0.02g 25 20
Injection Volume	: 25 uL
Data Filename	: MAH Peganum harmala 0.02g 25 2012 2 3 16.lcd
Method Filename	: tocopherols analysis hex isoprop 99.5 0.5.lcm
R[illegible]	[illegible]

Chromatogram

D [illegible]

MAH Peganum harmala 0.02g 25 2012 2 3 16 C:\...\LCsolution\data files imed\MAH Peganum harmala 0.02g 25 2012 2 3 16.lcd

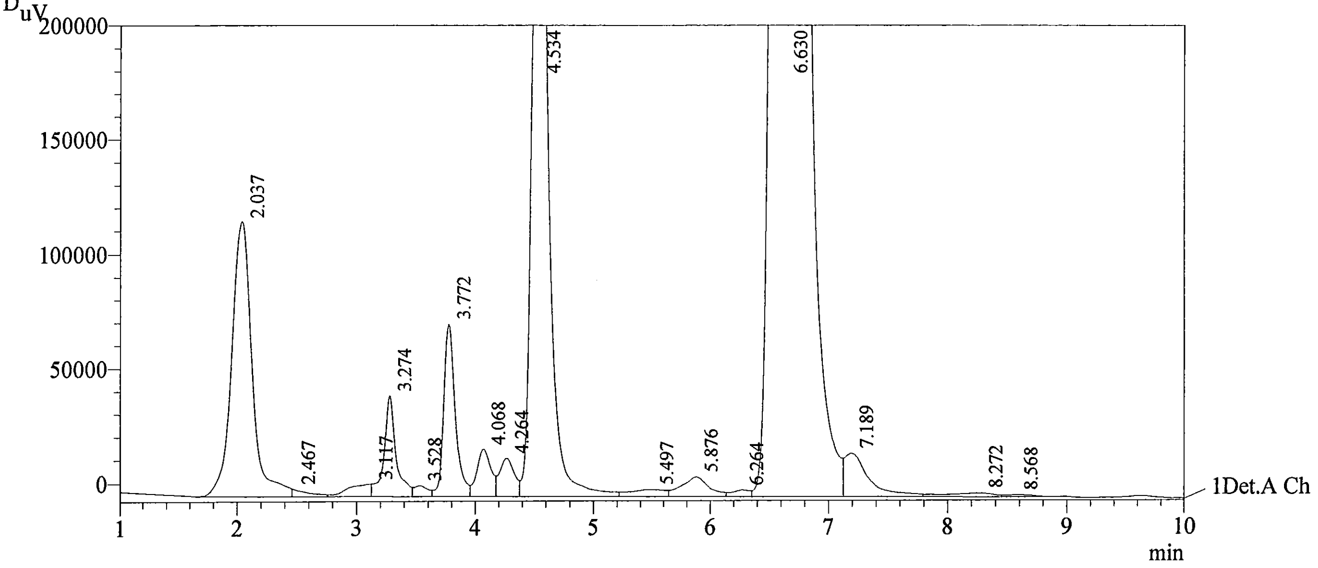

1 Det.A Ch1 / 295nm - 330nm

PeakTable

Detector A Ch1 295nm - 330nm

Peak#	Name	Ret. Time	Height	Area	Area %
1		2.037	120010	1432490	3.188
2		2.467	3166	28982	0.064
3		3.117	5333	71621	0.159
4		3.274	43610	307065	0.683
5		3.528	4667	37697	0.084
6		3.772	75115	516408	1.149
7		4.068	20312	168280	0.374
8		4.264	16513	142806	0.318
9		4.534	404999	3398216	7.562
10		5.497	2944	61832	0.138
11		5.876	8433	135001	0.300
12		6.264	2727	28674	0.064
13		6.630	3538507	38301620	85.227
14		7.189	18806	261153	0.581
15		8.272	1516	39377	0.088
16		8.568	688	9418	0.021
Total			4267345	44940640	100.000

Sample Information

Analyzed by	: Admin
Analyzed	: 2/1/2016 4:57:18 PM
Sample Type	: Unknown
Level #	: 1
Sample Name	: MOH hajji
Sample ID	:
Vial #	: 5
Injection Volume	: 1.00
Data File	: C:\GCMSsolution\Data\Project1\FAME\MOH hajji.qgd
Method File	: C:\GCMSsolution\Data\Project1\FAME\FAME-1,5 F O115 R 2-.qgm
Report File	:

Top Position

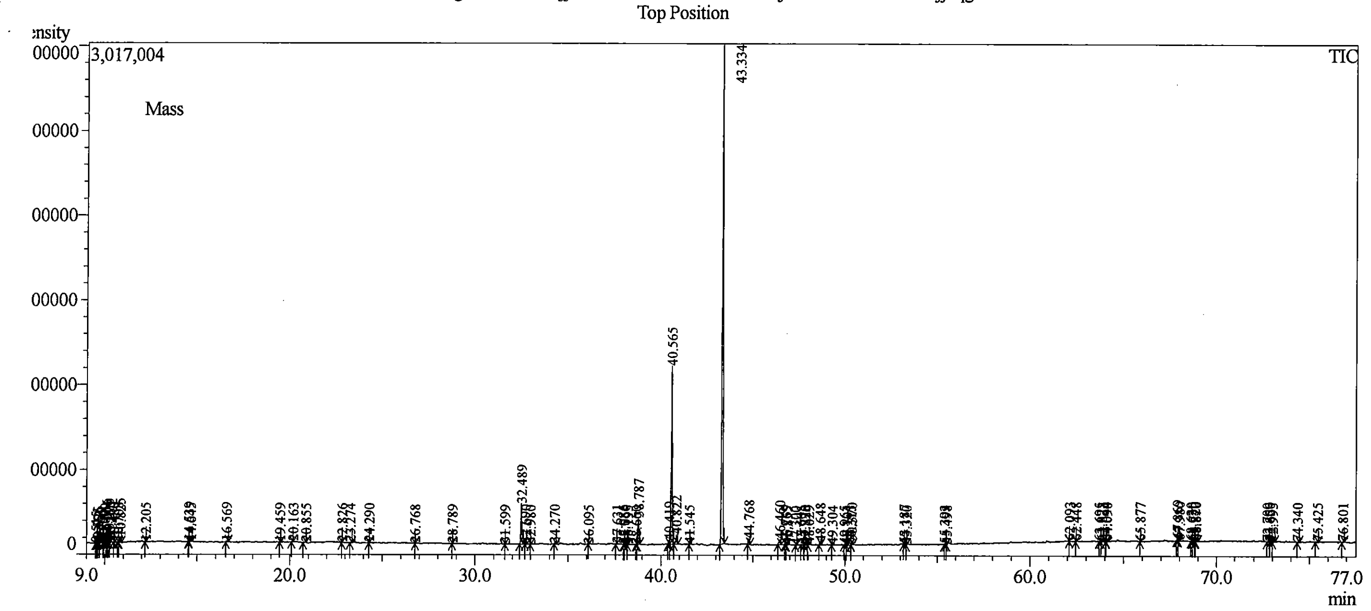

Peak Report TIC

Peak#	R.Time	Name	Base m/z	Area	Area%
1	9.515		167.60	10798	0.05
2	9.595		91.00	56276	0.27
3	9.626		91.00	69495	0.34
4	9.795		57.95	59050	0.29
5	9.899		471.85	14474	0.07
6	10.000		471.85	21106	0.10
7	10.096		91.00	59720	0.29
8	10.200		105.00	75133	0.36
9	10.260		105.00	24383	0.12
10	10.480		471.90	14125	0.07
11	10.708		105.00	16542	0.08
12	10.825		104.95	93060	0.45
13	12.205		471.90	11695	0.06
14	14.539		471.85	10343	0.05
15	14.645		471.90	30176	0.15
16	16.569		471.90	31024	0.15
17	19.459		471.85	9306	0.05
18	20.163		471.85	12052	0.06
19	20.855		471.90	24150	0.12
20	22.826		471.85	6601	0.03
21	23.274		471.85	26907	0.13
22	24.290		471.90	4975	0.02
23	26.768		471.90	5124	0.02
24	28.789		471.90	4721	0.02
25	31.599		471.85	3353	0.02
26	32.489	C16	74.00	989942	4.80
27	32.690		471.85	15842	0.08
28	32.980		471.85	6992	0.03
29	34.270		471.85	23428	0.11
30	36.095		470.95	5708	0.03
31	37.631		471.85	16419	0.08
32	37.985		470.95	11872	0.06
33	38.060		471.85	17992	0.09
34	38.195		471.90	12072	0.06
35	38.660		471.85	8075	0.04
36	38.787	C18	74.00	624049	3.03
37	40.419		54.95	101344	0.49
38	40.565	C18|1 w9	54.95	4358992	21.14
39	40.822	C18|1 w7	54.95	248236	1.20
40	41.545	[illegible]	471.85	7923	0.04
41	43.334	C18|2 w6	67.00	12473085	60.49
42	44.768	C20	74.00	132392	0.64
43	46.460	C18|3 w3	79.00	156400	0.76
44	46.769		472.80	11486	0.06
45	46.855		472.90	12334	0.06

Peak#	R.Time	Name	Base m/z	Area	Area%
46	47.300		470.95	18505	0.09
47	47.605		471.85	15784	0.08
48	47.770		471.85	6447	0.03
49	47.825		471.85	5939	0.03
50	48.029		472.85	11685	0.06
51	48.648	$C_{40}H_{82}$	57.00	63740	0.31
52	49.304		471.85	3773	0.02
53	49.965		471.85	24378	0.12
54	50.104		471.85	17392	0.08
55	50.315		470.95	6829	0.03
56	50.370	C22	74.00	77582	0.38
57	53.187		471.85	7269	0.04
58	53.320		470.95	4391	0.02
59	55.393		471.85	21724	0.11
60	55.498		471.85	4245	0.02
61	62.093		471.85	4663	0.02
62	62.448		471.85	5760	0.03
63	63.695		470.95	21884	0.11
64	63.828		471.85	26928	0.13
65	64.044		471.85	8450	0.04
66	64.090		471.85	4881	0.02
67	65.877		471.85	9978	0.05
68	67.869		473.85	24035	0.12
69	67.980		470.95	14590	0.07
70	68.620		472.85	18056	0.09
71	68.730		470.95	31070	0.15
72	68.820		472.85	11116	0.05
73	68.870		471.85	39350	0.19
74	72.700		471.85	30742	0.15
75	72.850		471.85	40436	0.20
76	72.995		471.85	33884	0.16
77	74.340		471.85	18317	0.09
78	75.425		471.85	35583	0.17
79	76.801		470.95	21618	0.10
				20620196	100.00

KING SAUD UNIVERSITY
COLLEGE OF SCIENCE / CHEMISTRY DEPARTMENT

Sample Information

Acquired by	: Dr. Nehdi I. A
Sample Name	: SSE Peganum harmala 0.02g 25 2012 2 3 16
Sample ID	: SSE Peganum harmala 0.02g 25 20
Injection Volume	: 25 uL
Data Filename	: SSE Peganum harmala 0.02g 25 2012 2 3 16.lcd
Method Filename	: tocopherols analysis hex isoprop 99.5 0.5.lcm
[illegible]	[illegible]

Chromatogram

SSE Peganum harmala 0.02g 25 2012 2 3 16 C:\LabSolutions\LCsolution\data files imed\SSE Peganum harmala 0.02g 25 2012 2 3 16.lc

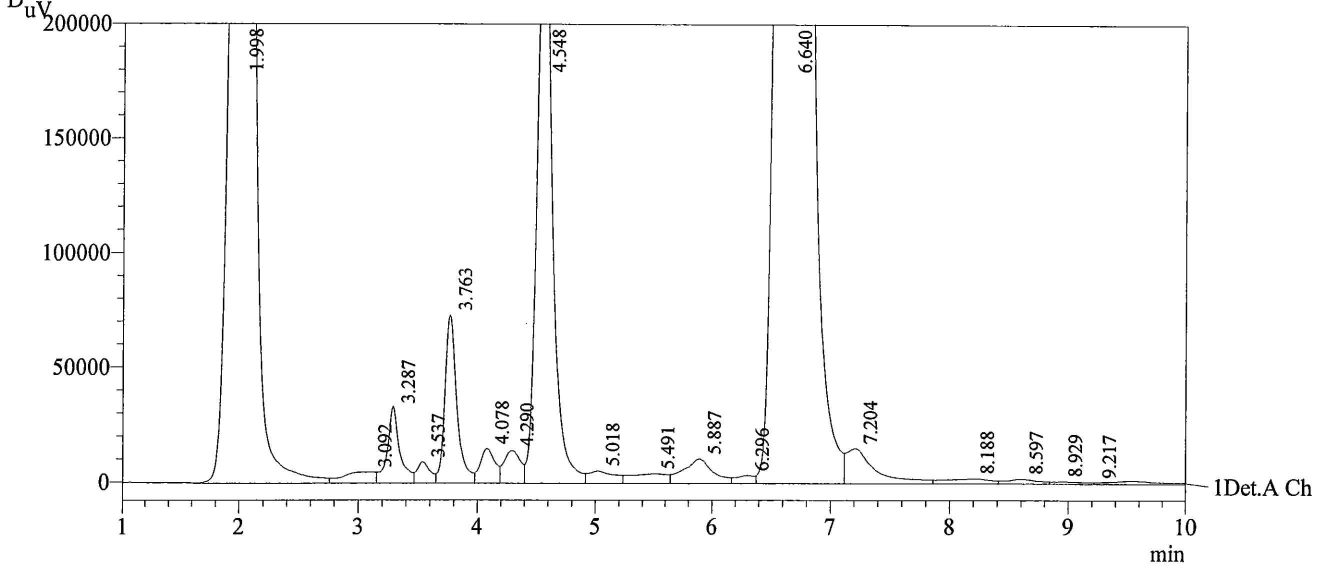

1 Det.A Ch1 / 295nm - 330nm

PeakTable

Detector A Ch1 295nm - 330nm

Peak#	Name	Ret. Time	Height	Area	Area %
1		1.998	914704	9130423	20.636
2		3.092	4726	87613	0.198
3		3.287	33340	242201	0.547
4		3.537	9187	69142	0.156
5		3.763	73167	513559	1.161
6		4.078	14966	131569	0.297
7		4.290	14087	137542	0.311
8		4.548	269506	2319740	5.243
9		5.018	5287	81169	0.183
10		5.491	4035	91412	0.207
11		5.887	10593	185111	0.418
12		6.296	3403	37746	0.085
13		6.640	2848067	30789823	69.591
14		7.204	15269	259192	0.586
15		8.188	2098	56897	0.129
16		8.597	1972	36585	0.083
17		8.929	884	15811	0.036
18		9.217	524	58689	0.133
Total			4225815	44244225	100.000

Sample Information

Analyzed by	: Admin
Analyzed	: 2/1/2016 3:36:07 PM
Sample Type	: Unknown
Level #	: 1
Sample Name	: SSE hajji
Sample ID	:
Vial #	: 4
Injection Volume	: 1.00
Data File	: C:\GCMSsolution\Data\Project1\FAME\SSE hajji.qgd
Method File	: C:\GCMSsolution\Data\Project1\FAME\FAME-1,5 F O115 R 2-.qgm
Report File	:

Top Position

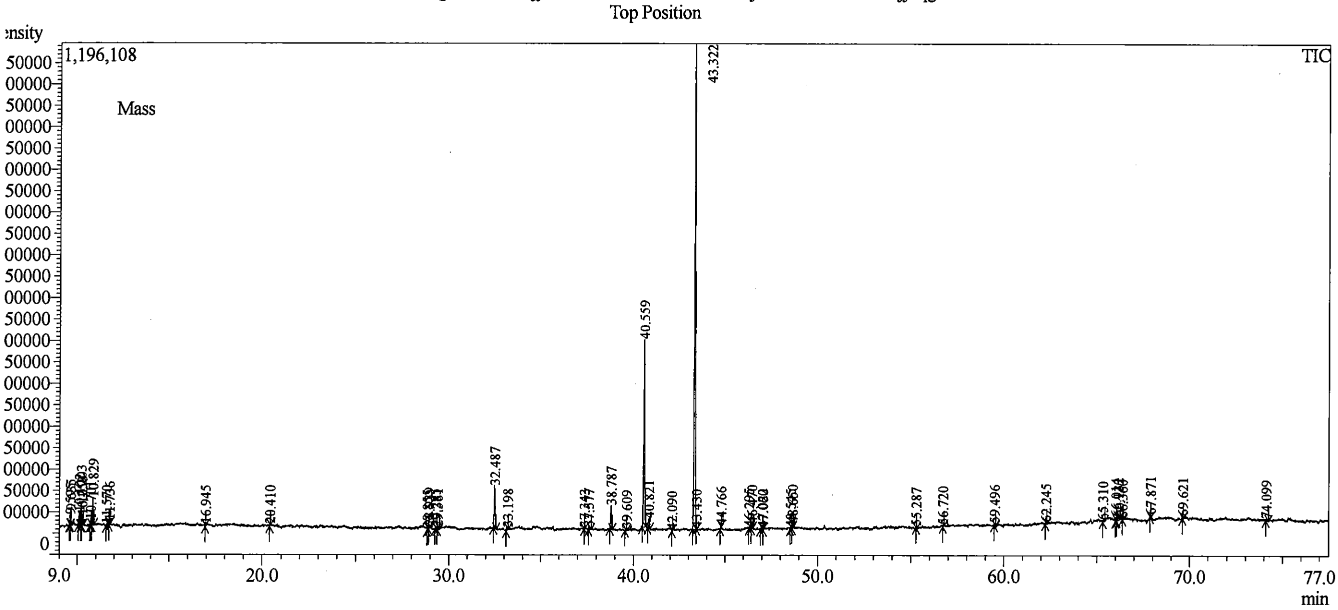

Peak Report TIC

Peak#	R.Time	Name	Base m/z	Area	Area%
1	9.595		91.00	78125	0.94
2	9.635		91.00	61491	0.74
3	10.102		91.00	76129	0.91
4	10.203		105.00	125090	1.50
5	10.258		105.00	56778	0.68
6	10.701		105.05	33228	0.40
7	10.829		105.00	162173	1.94
8	11.570		471.90	29242	0.35
9	11.736		105.00	33556	0.40
10	16.945		471.90	5490	0.07
11	20.410		471.90	8720	0.10
12	28.855		471.95	12356	0.15
13	28.939		471.90	8476	0.10
14	29.285		471.90	14441	0.17
15	29.381		472.90	27183	0.33
16	32.487	C16	74.00	401116	4.81
17	33.198		471.05	24682	0.30
18	37.343		471.90	3766	0.05
19	37.577		471.90	32280	0.39
20	38.787	C18/0	74.00	216258	2.59
21	39.609		471.90	16310	0.20
22	40.559	C18/1 w9	55.00	1770553	21.21
23	40.821	C18/1	55.00	86700	1.04
24	42.090		471.90	5182	0.06
25	43.322	C18/2 w6	67.00	4710595	56.43
26	43.430	Apiol	471.90	1382	0.02
27	44.766	C20	73.95	19033	0.23
28	46.295		471.90	16752	0.20
29	46.470	C18/3	79.00	42411	0.51
30	47.022		472.90	23219	0.28
31	47.060		471.90	3535	0.04
32	48.545		471.90	11080	0.13
33	48.650	C40[illegible] [illegible]	57.00	25249	0.30
34	55.287		471.90	4578	0.05
35	56.720		306.90	8942	0.11
36	59.496		471.90	14451	0.17
37	62.245		471.90	13051	0.16
38	65.310		471.90	18033	0.22
39	66.014		473.90	30574	0.37
40	66.132		471.90	64084	0.77
41	66.360		471.90	7861	0.09
42	67.871		471.90	10167	0.12
43	69.621		471.90	9783	0.12
44	74.099		471.90	23232	0.28
				8347337	100.00

C_{22} ?
